# An EEG study of neurophysiological markers of cognitive reserve

**DOI:** 10.1002/alz.084989

**Published:** 2025-01-09

**Authors:** Osamu Katayama, Yaakov Stern, Christian G Habeck, Annabell Coors, Sangyoon Lee, Kenji Harada, Keitaro Makino, Kouki Tomida, Masanori Morikawa, Ryo Yamaguchi, Chiharu Nishijima, Yuka Misu, Kazuya Fujii, Takayuki Kodama, Hiroyuki Shimada

**Affiliations:** ^1^ National Center for Geriatrics and Gerontology, Obu City, Aichi Japan; ^2^ Columbia University Vagelos College of Physicians and Surgeons, New York, NY USA; ^3^ Japan Society for the Promotion of Science, Chiyoda–ku, Tokyo Japan; ^4^ Kyoto Tachibana University, Yamashina–ku, Kyoto Japan; ^5^ Cognitive Neuroscience Division, Columbia University Vagelos College of Physicians and Surgeons, New York, NY USA

## Abstract

**Background:**

Cognitive reserve (CR) is a property of the brain trait that allows for better–than–expected cognitive performance, relative to the degree of brain change over the life course. However, the neurophysiological markers of CR require further investigation. Electroencephalography (EEG) may provide an appropriate neurophysiological marker of CR. Therefore, we tested whether activity in the dorsal attention network (DAN) and the ventral attention network (VAN), as measured during resting–state EEG, moderates the relationship between hippocampal volume and episodic memory.

**Method:**

Participants from the National Center for Geriatrics and Gerontology–Study of Geriatric Syndromes, were included; all underwent determined hippocampal volume using MRI and episodic memory was measured using word lists. After testing the effect of hippocampal volume on memory performance using multiple regression analysis, we evaluated the interactions between hippocampal volume and DAN and VAN network activity. We used the Johnson–Neyman technique to quantify the moderating effects of the DAN and VAN network activity on the relationship between hippocampal volume and word list memory, and to identify specific ranges of the DAN and VAN network activity with significant hippocampal–memory associations.

**Result:**

A total of 449 participants were included in this study. We found significant moderation of DAN with a slope of β = ‐0.0001 (95% CI: ‐0.0002; ‐0.00001, p = 0.041) and VAN was significant, with a slope of β = 0.0001 (95% CI: 0.00001; 0.0003, p = 0.032). We found that larger hippocampal volume was associated with better memory performance, and this association became stronger the lower the DAN activity. However, when the DAN activity was > 944.9, the hippocampal volume was no longer significantly related to word list memory performance. For VAN, we found higher hippocampal volume was more strongly associated with better memory performance when VAN activity was higher. However, when the VAN activity was < 914.6, the hippocampal volume was no longer significantly associated with word list memory.

**Conclusion:**

Our results suggest that attention network helps maintain memory performance in the face of age related structural decline, meeting criteria for a neural implementation of cognitive reserve.

